# Is impairment of facial emotion recognition independent of cognitive dysfunction in multiple sclerosis?

**DOI:** 10.1007/s10072-024-07314-0

**Published:** 2024-01-22

**Authors:** Yagmur Simge Sever Aktuna, Asli Koskderelioglu, Neslihan Eskut, Atalay Aktuna

**Affiliations:** 1 Neurology Department, University of Health Sciences, Izmir Bozyaka Education and Research Hospital, 35170 Izmir, Turkey; 2 Department of Public Health, Ministry of Health, Bornova District Health Directorate, 35030 Bornova, Izmir, Turkey

**Keywords:** Cognitive dysfunction, Empathy, Facial emotion recognition, Multiple sclerosis, Social cognition

## Abstract

**Background:**

Emotions expressed on the face play a key role in social cognition and communication by providing inner emotional experiences. This study aimed to evaluate facial emotion identification and discrimination and empathy abilities in patients with MS and whether it is related to cognitive dysfunction.

**Methods:**

One hundred twenty patients with relapsing–remitting MS and age- and sex-matched 120 healthy controls were enrolled in the study. All the subjects were evaluated with the Facial Emotion Identification Test (FEIT), Facial Emotion Discrimination Test (FEIDT), and Empathy Quotient (EQ). We used the Beck Depression Inventory (BDI) for depression and detailed cognitive tests, including the Montreal Cognitive Assessment (MoCA), the Symbol Digit Modalities Test (SDMT), and the Paced Auditory Serial Addition Test (PASAT). The quality of life was assessed with Multiple Sclerosis Quality of Life-54 (MSQL-54).

**Results:**

Patients with MS were 37.6 ± 9.5 years old, had a mean disease duration of 8.8 ± 6.6 (8–28) years, and a mean EDSS score of 1.6 ± 1.3 (0–4.5). We found significant differences in the identification of facial emotions, discrimination of facial emotions, and empathy in MS patients compared to controls (*p* < 0.05). Especially the recognition of feelings of sadness, fear, and shame was significantly lower in MS patients. The multivariate logistic regression analysis showed low SDMT and FEIDT scores which showed an independent association with MS.

**Conclusions:**

Our findings indicate that facial emotion recognition and identification deficits are remarkable among patients with MS and emotion recognition is impaired together with and independently of cognitive dysfunction in MS patients.

## Introduction

Cognitive dysfunction is identified in all multiple sclerosis (MS) subtypes at any stage of the disease process. Several studies established that 40–65% of MS patients have cognitive dysfunction; however, in daily practice, it is detected in only 5% [1]. The cognitive domains predominantly impaired in patients with MS include information processing speed, memory, and executive functions. Although these domains have been well investigated in MS, more attention needs to be provided to social cognition (SC), defined as a multidimensional construct that encircles the theory of mind (ToM), emotional recognition (ER), and empathy [2].

It has recently been recognized that social cognitive functions are also crucial in MS. Eventually, social cognition became a particular interest in MS. Consequently, the primary research on MS patients targets reduced physical ability and cognitive impairment. Nevertheless, social cognition profoundly affects the quality of life, interpersonal communication skills, and cognitive dysfunction.

An essential component of social cognition includes facial expressions and their emotional significance, which is one of the critical elements of social interaction. Social cognition deficits are associated with difficulties in understanding the emotions of those who communicate, leading to low social competence [3], poor communication skills [4], and poor quality of life [5]. In the last two decades, the ability to recognize emotional facial expressions has been investigated, which are known to affect the social functioning of MS patients. However, limited studies examined the relationship between emotion recognition, cognitive functions, and quality of life in MS patients.

Most emotion recognition studies have focused on facial expressions; however, numerous methods of evaluating social cognition exist. On the other hand, a photograph series consisting of 110 black and white digital photographs of 35 mm size, embodying 6 different emotions, was created by Paul Ekman in 1976 [6]. Moreover, many tests, including the Facial Emotion Identification Test (*FEIT*) and the Facial Emotion Discrimination Test (*FEIDT*), were subsequently developed to evaluate emotion recognition based on Ekman’s research. In 1993, Kerr and Neale used FEIT and FEIDT tests in schizophrenia patients to determine how facial emotion perception ability deteriorated [7].

Our hypothesis was that MS patients would have impairments in social cognition with a decreased facial emotion recognition ability independent of the cognitive dysfunction. In this context, we aimed to evaluate social cognition using FEIT, FEIDT, and EQ tests in MS patients and to examine the relationship between social cognition deficits and cognitive dysfunction, affective status, and quality of life.

## Material and methods

### Participants

**Fig. 1:**
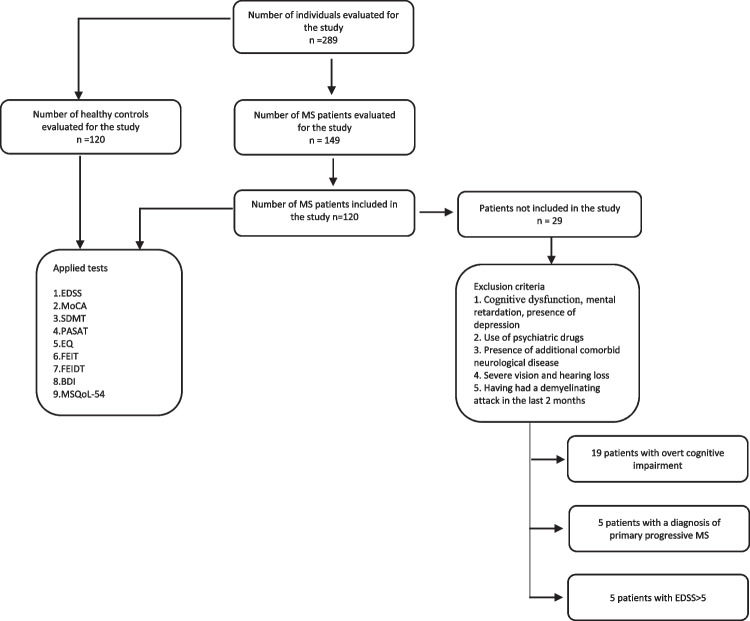
Flowchart of the participants included in the study

This study was carried out between December 2020 and June 2021 at the neurology department of Izmir Bozyaka Education and Research Hospital. A priori sample size was calculated as 200 with 100 participants in each group, using G*Power (Ver. 3.1.9.4, Dusseldorf University, Germany) for alpha probability error of 0.05, power of 0.80, allocation ratio of 1, and effect size of 0.40 which is determined based on data in the literature and clinical opinion for all social cognitional outcomes. In order to increase the power of the study and to perform possible subgroup analyses, a total of 240 participants, 120 in each group, were targeted, which would be above the a priori sample size. This study involved relapsing–remitting subtype of MS patients between 18 and 55 years old who had at least primary school education. We evaluated 149 consecutive patients from the university hospital’s MS outpatient clinic and included 120 of them. Age-, sex-, and education level-matched 120 volunteers were recruited from the community who served as healthy controls (HC). We excluded participants if they had a neurological (other than MS in the patient group) disease, psychiatric illness, including current depression, current use of antipsychotic or antidepressant medications, a relapse or steroid treatment in the previous 2 months, cognitive dysfunction documented in clinical follow-ups which resulted in impairment in daily activities or mental retardation, and a significant visual or auditory impairment that would interfere with test instructions. Figure 1 displays the flowchart of the study.

All participants provided written informed consent before approaching the study, which the ethics committee approved of our institution (decision no:03, date: 11.11.2020).

### Clinical assessment

We collected the demographic data throughout the study period with face-to-face interviews. Age, gender, education level, disease duration, annualized relapse rate, comorbid diseases, and current disease-modifying treatment were recorded. All the participants underwent the neurological examination by the same neurologist to generate an Expanded Disability Scale Score (EDSS) score [8] and complete all measurements and clinical tests.

### Social cognition assessment

#### Emotion recognition assessment

##### Facial Emotional Identification Test (FEIT)

This test was developed by Kerr and Neale [7] in 1993, and its validity and reliability study for Turkish culture was performed by Erol et al. [9]. The test consists of black and white photographs of the faces of 19 different people, each embodying one of six other emotions (happiness, sadness, anger, surprise, fear, shame). Of these 19 photographs, 15 depict negative emotions (sadness, anger, fear, and shame), while four depict positive ones (happiness and surprise). The participant is given a 19-item answer form, each consisting of six emotion options, and each photo is shown on the computer screen for 15 s with a 10-s gap between them. After each stimulus, the participant was required to mark on the form which of the six emotions shown in the picture was chosen. The total test score was calculated as the number of accurate answers (0–19).

##### Facial Emotional Discrimination Test (FEIDT)

Validity and reliability studies of this test in our country were performed [9]. This test consists of 30 pairs of black and white photographs containing the six emotions shown at FEIT. The participant is asked to distinguish whether the emotion expressed on the two faces depicted in each pair of pictures is the same or different. For this study, each participant was presented with these photographs in the same order as the FEIT and was given a 30-item response form with two choices: “same” and “different.” After each picture, the participant was asked to mark their answer on the response form. The score was calculated as the number of correct answers (0–30).

#### Empathy assessment

##### Empathy Quotient (EQ)

The scale aims to evaluate empathy within the framework of social understanding. EQ, for assessing empathy 40 and 20 distracting items to prevent the person from focusing on the purpose of the test. A Likert-type scale with four options was used (1 = strongly disagree, 2 = somewhat disagree, 3 = partly agree, 4 = strongly agree). During scoring, only 40 questions measuring empathy are calculated. The least empathetic two responses are given 0 points, the most empathetic two responses, and the second empathic responses 1 point. The highest score is 80. “I strongly agree” in some of the items and “strongly disagree” in others indicate an empathetic response. There is no cutoff score for the test [10].

### Cognitive assessment

The participants were administered the MoCA test, Symbol Digit Modalities Test–oral version (SDMT), and Paced Auditory Serial Addition Test (PASAT) tests for detailed cognitive evaluation [11–13]. SDMT was used to evaluate visual information processing speed from cognition domains, and the PASAT test was used for auditory information processing speed and working memory assessment. The MoCA test is a screening instrument that allows cognitive measurement including attention, concentration, short time memory, executive functions, delayed recalling language, visuo-spatial abilities, conceptual thinking, calculation, and orientation. Although the MoCA test is not a specific cognitive evaluation among MS patients, its combined use along with SDMT is recommended [14].

### Depression assessment

Beck Depression Inventory (BDI-I) is a widely used self-assessment tool consisting of 21 questions to determine the severity of depressive symptoms [15]. The score varies between 0 and 63. In detail, 0–10 points are interpreted as usual, 11–17 points as mild depression, 18–23 points as moderate depression, and 24 points and above as severe depression. The cutoff point of the scale is 17.

### Quality of life assessment

Quality of life was assessed by the MS-specific quality of life-54 (MSQL-54) [16, 17].

### Statistical analysis

All statistical data were analyzed using SPSS version 24.0 (SPSS, Chicago, IL, USA). Descriptive results were given as mean, standard deviation (SD), median, minimum, and maximum values for continuous data, and as numbers and percentages for categorical data. Patients were compared to healthy controls regarding demographic characteristics, cognitive and neuropsychological tests, and social cognition. Data normality was analyzed using the Kolmogorov–Smirnov test, the Shapiro–Wilk test, and histogram plots. Student’s *t*-test or Mann–Whitney *U* test was used according to the data normality to compare the groups. The Chi-square test was used to compare the gender distribution of patients and healthy controls. Pearson or Spearman correlation analysis was used to calculate the correlations between patients’ FEIT, FEDT, EQ scores and disease duration, annualized attack rate, EDSS scores, cognition, depression status, and quality of life according to the normality of data. Variables with significant differences between the patient and HC as a result of bivariate analyses were included in the backward stepwise logistic regression model and multivariate analysis was performed, and it was determined which variables were independently associated with MS disease when compared with HC. Differences were considered significant at a *p*-value of < 0.05.

## Results

One hundred twenty healthy controls (HCs) and 149 RRMS patients were recruited. Twenty-nine patients were excluded due to having either prominent cognitive impairment, primer progressive MS, or severe neurologic disability (EDSS > 5). Patients with MS were compared to age, sex, and education level matched HCs. The flowchart of the study is expressed in Fig. 1. The demographic data and clinical parameters are summarized in Table 1.

**Table 1:** Clinical and demographic characteristics

	Healthy controls	RRMS patients	*p*
Group size (*n*)	120	120	
Female/male: *n* (%)	73/47 (60.8)	78/42 (65)	0,504^a^
Age (years) (mean ± SD)	36.13 ± 9.38	37.64 ± 9.51	0,215^b^
Education level (years) (mean ± SD)	12.58 ± 3.91	11.83 ± 3.86	0,075^b^
EDSS (0–10) [median (min–max)]		1.0 (0–4.5)	
Disease duration (years) (mean ± SD)		8.8 ± 6.6	
ARR (mean ± SD)		1.08 ± 2.2	

*SD* standard deviation, *EDSS* Expanded Disability Status Scale, *ARR* annualized relapse rate

Statistical test conducted: ^a^Pearson chi-square, ^b^Student *t*-test

*P* < 0.05 was considered statistically significant

### Comparison of MS patients with healthy controls (HC)

**Fig. 2:**
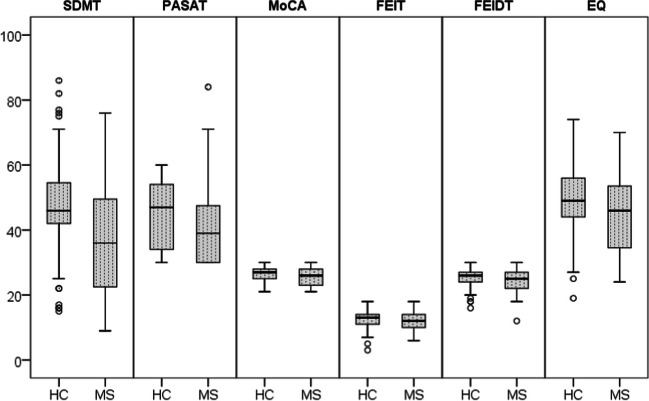
Comparison of MS and HCs in social cognition and other cognitive domains. Abbreviations: HC, healthy control; MS, multiple sclerosis; SDMT, Symbol Digit Modalities Test; PASAT, Paced Auditory Serial Addition Test; MoCA, Montreal Cognition Assessment; EQ, Empathy Quotient; FEIDT, Facial Emotion Discrimination Test; FEIT, Facial Identification Test

**Fig. 3:**
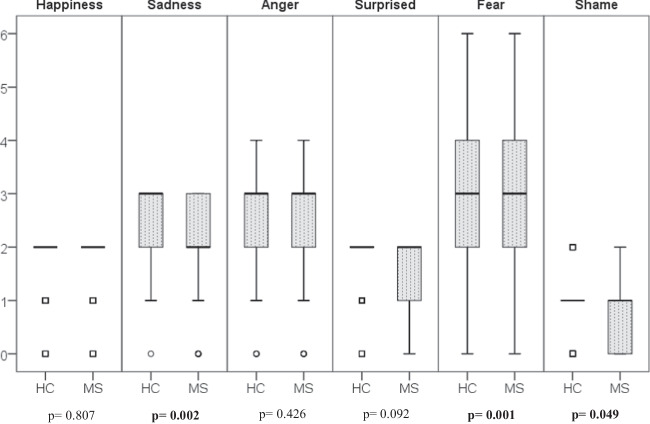
Distribution of FEIT test results of MS patients and HC group. Abbreviations: HC, healthy control; MS, multiple sclerosis. Statistical test conducted: Mann Whitney *U* test, statistical significance: *p* < 0.05. MS patients and HCs were compared in terms of recognition ability of six facial emotions, a statistically significant difference was found in the recognition of “sadness,” “fear,” and “shame” emotions

The cognitive performance was worse in MS patients than HCs in both social and other cognitive domains (Fig. 2 and Table 2). BDI test scores in MS patients were significantly higher than in healthy controls. MS patients and HCs were compared regarding the recognition ability of six facial emotions. The recognition of “sadness,” “fear,” and “shame” feelings statistically differed in MS patients compared to HCs (Fig. 3).

**Table 2:** Data of test scores applied to MS patient group and healthy controls and comparison of the two groups

		Mean	Standard deviation	Median	Maximum	Minimum	*p*
FEIT	HC	12.79	2.56	13.00	18.00	3.00	**0.001**
	MS	11.67	2.74	12.00	18.00	6.00	
FEIDT	HC	25.68	2.58	26.00	30.00	16.00	** < 0.001**
	MS	24.31	3.09	25.00	30.00	12.00	
EQ	HC	49.10	9.97	49.00	74.00	19.00	0.003^*^
	MS	44.92	11.55	46.00	70.00	24.00	
MoCA	HC	26.61	2.29	27.00	30.00	21.00	**0.010**
	MS	25.65	2.78	26.00	30.00	21.00	
PASAT	HC	44.95	10.50	47.00	60.00	30.00	**0.001**
	MS	40.53	10.94	39.00	84.00	30.00	
SDMT	HC	47.61	13.77	46.00	86.00	15.00	** < 0.001**
	MS	35.86	16.84	36.00	76.00	9.00	
BDI	HC	10.53	8.77	8.50	41.00	0.00	**0.042**
	MS	13.83	11.43	11.00	48.00	0.00	

*MoCA* Montreal Cognitive Assessment Test, *PASAT* Paced Auditory Serial Addition Test, SDMT Symbol Digit Modalities Test, *EQ* Empathy Quotient, *FEIDT* Facial Emotion Discrimination Test, *FEIT* Facial Identification Test, *BDI* Beck Depression Inventory

*P* < 0.05 was considered statistically significant. Statistically significant *p* values are shown in bold

Statistical test conducted: Mann Whitney *U* test, *Student *t*-test

### Evaluation of the relationship between social cognition, demographic features, and disease parameters

Patients with MS showed a moderate negative correlation between FEIT test scores and age (*r* =  − 0,355, *p* < 0,001) and a low negative correlation between FEIT and EDSS scores (*r* =  − 0,267, *p* = 0,003). A low-level negative correlation between FEIDT test scores and age was found (*r* =  − 0.242, *p* = 0.008). However, FEIDT scores showed a moderate positive correlation with education level (*r* = 0.311, *p* = 0.001). No correlation was found between EQ scores and age, education level, ARR, disease duration, and EDSS.

### Evaluation of the relationship between social cognition and other cognitive tests

In the patient group, a positive and significant correlation was found between the MoCA test scores and other cognitive test scores (PASAT, SDMT) as well as social cognition test scores (FEIT, FEIDT, EQ) (Table 3). We found a positive low-level correlation between SDMT, FEIT, and FEIDT test scores. The PASAT scores showed a positive low-level correlation with FEIT scores (Table 3).

**Table 3:** Relationship between cognitive test scores and FEIT, FEIDT, and EQ scores in MS patients

		MoCA	SDMT	PASAT	EQ	FEIT	FEIDT
MoCA	*r*	1.000	**0.476**	**0.468**	**0.200**	**0.332**	**0.252**
	*p*		** < 0.001**	** < 0.001**	**0.029**	** < 0.001**	**0.006**
SDMT	*r*		1.000	**0.310**	0.139^*^	**0.266**	**0.269**
	*p*			**0.001**	0.130^*^	**0.003**	**0.003**
PASAT	*r*			1.000	0.030	**0.265**	0.126
	*p*				0.743	**0.003**	0.169
EQ	*r*				1.000	0.022	0.151
	*p*					0.811	0.100
FEIT	*r*					1.000	**0.241**
	*p*						**0.008**
FEIDT	*r*						1.000
	*p*						

*MoCA* Montreal Cognitive Assessment Test, *PASAT* Paced Auditory Serial Addition Test, *SDMT* Symbol Digit Modalities Test, *EQ* Empathy Quotient, *FEIDT* Facial Emotion Discrimination Test, *FEIT* Facial Identification Test

*P* < 0.05 was considered statistically significant. Statistically significant *p* values are shown in bold

Statistical test conducted: Spearman correlation test, *Pearson correlation test

### Evaluation of the relationship between social cognition and depression

In the MS group, a negative and low-level significant correlation was found between the BDI test scores and both EQ and FEIT scores (*r* =  − 0.295, *p* = 0.001, *r* =  − 0.186, *p* = 0.042, respectively).

### Evaluation of the relationship between social cognition and quality of life

In MS patients, a positive and low-level significant correlation was found between the MSQL-54-MHC (Mental Health Composite) and EQ scores (*r* = 0.274, *p* = 0.002).

### The evaluation of social cognition, cognition, and depression related to MS disease by multivariate analysis

We performed the multivariate backward stepwise logistic regression analysis according to the significantly different variables identified between MS patients and HCs in the pairwise analysis. First, we included the variables, along with MoCA, PASAT, SDMT, BDI, FEIT, FEIDT, and EQ tests, in the model; afterward, we started the analysis. The analysis was carried out in 6 steps in total, and PASAT, MoCA, BDI, FEIT, and EQ were excluded from the model, respectively, with one variable in each step. The final model formed in the sixth step determined that low SDMT and FEIDT scores were associated with MS when the HCs were taken as a reference. Estimates of the created model were not different from the actual situation (Hosmer & Lemeshow *p* > 0.05). We observed that 20% of MS patients could be explained by the independent variables in the final model (Nagelkerke *R*^2^ = 0.20) (Table 4).

**Table 4:** MS disease-associated tests, backward stepwise logistic regression analysis final model

	B (SE)	Wald	OR (%95 GA)	*p*
SDMT	− 0.046 (0.10)	22.36	0.95 (0.94–0.97)	** < 0.001**
FEIDT	− 0.137 (0.53)	6.70	0.87 (0.79–0.97)	**0.010**

*SDMT* Symbol Digit Modalities Test; *FEIDT* Facial Emotion Discrimination Test

*R*^2^ = 0.15 (Cox & Snell), 0.20 (Nagelkerke), *X*^2^(8) = 14.26, *p* > 0.05 (Hosmer & Lemeshow)

*P* < 0.05 was considered statistically significant. Statistically significant *p* values are shown in bold

## Discussion

In this study, among the 6 emotions evaluated, especially the recognition of feelings of sadness, fear, and shame was significantly lower in MS patients than in HCs. Cecchetto et al. found that the recognition of all emotion subgroups was impaired in MS patients [18]. However, several studies show a selective deterioration in recognizing negative emotions, especially anger, fear, and sadness [19, 20]. The reason for the selective impairment in recognition of negative emotions is the relatively more straightforward processing of positive emotions than negative emotions, the low sensitivity of MS patients to aversive stimuli, and the hypoactivation of cortical areas that process negative emotions [21].

There are inconsistencies in studies examining the relationship between emotion recognition and educational status [18, 22]. We have found an association between high performance in distinguishing facial emotions and the length of the education period. Chalah et al. explain the relationship between social cognition and education by referring to the “cognitive reserve hypothesis,” in which a higher education level is considered one of the protective factors against cognitive decline, including social cognition, despite MS-related brain damage [2]. Our findings support this hypothesis because there was no relationship between emotion recognition and education level in the healthy control group in this study. However, further studies should support this finding by MRI data.

In this study, we found that the decrease in the FEIT score in MS patients was associated with advanced patient age, long disease duration, and a high EDSS score. Jehna et al. stated that disorders in emotion recognition might occur early in the disease [22]. Another study conducted in 2003 showed that impairment in emotion recognition is not limited to patients with progressive disease forms [23, 24]. These results in the literature are in line with our findings. On the other hand, Henry et al. reported that MS patients with physical disabilities exhibited significant deficits in emotion recognition, regardless of their disability [19]. Similarly, Banati et al. did not find a substantial relationship between emotion recognition and patients’ EDSS scores. However, they interpreted that mentalization disorders were more severe in patients with a long course of the disease, as the increase in the duration of the illness and disability caused a decrease in social cognition [25].

We have shown that EQ scores were significantly lower in MS patients than in HCs. Although many studies have found that MS patients have disturbed empathy functions [25–28], interestingly, RRMS patients describe themselves as inclined to share the emotional states of others and to understand the conditions of others [24, 26]. This is due to the fact that the questionnaires used in these studies were created to evaluate the level of empathy that individuals attributed to themselves by the authors. In addition, it is assumed that the deterioration in TOM, which is one of the components of social cognition, causes patients to evaluate themselves as more empathetic than they are. The emotional stress associated with the disease causes a more focused emotional processing, resulting in a higher empathy estimate [29]. Recent research on MS has described a decrease in empathy even in the early stages of the disease [27, 30], which is associated with the involvement of white and grey matter, particularly in the frontal lobes [31–34]. The current study found no relationship between empathy and disease duration and EDSS score. Similarly, in a study in Brazil in 2016 involving 34 MS patients and 34 controls, MS patients showed lower levels of empathy regardless of disease duration or neurological disability [26].

A relationship between decreased cognitive function and low scores in all social cognition tests in MS patients was found in our study. These findings were compatible with the data obtained in previous studies [27, 35–39]. However, several studies concluded that MS patients suffered social cognition problems independent of cognitive decline [25, 28, 40, 41]. Palermo et al. assumed that attention control might be necessary to distinguish facial emotions. Therefore, cognitive dysfunctions may also affect emotion recognition abilities [42].

Similarly, we enrolled MS patients without overt cognitive deficits in this study. We determined that social cognition deficits were remarkable in MS patients. In addition, in the backward stepwise logistic regression analysis, SDMT and FEIDT scores in the final model were found to be associated with MS disease. These results suggest that social cognition, especially recognizing facial emotions, may be impaired independently of cognitive function in MS patients and that social cognition in MS may deteriorate even before cognitive impairment begins. Social cognition abilities may worsen over time of the severity of cognitive impairment.

A relationship between a depressive mood, disturbed empathy, and impaired recognition of facial emotions among MS patients was found in the current study. The relationship between MS disease and BDI test score disappeared in the backward stepwise logistic regression model. These results suggest that the effect of depression symptoms on MS patients does not primarily react to impaired social cognition and other cognitive functions.

There have been inconsistent results in the literature arguing the relationship between depression and social cognition. It is thought that these differences may have led to different interpretations due to the difference in the depression levels of the patients included in the study and the tendency of MS patients to over-identify negative emotions and label the pictures shown as less happy.

We found that a lower empathy level was associated with a worse quality of life mental composite. However, no association was found between recognizing and distinguishing facial emotions and the quality of life. In line with our findings, a study found no association between quality of life and social cognition [43]. Researchers should interpret this negative result carefully by taking into account the unique characteristics of the scale and that the scale is a valid tool for evaluating the disease-related quality of life. However, they thought that the fact that motor disability is more physically restrictive in the patient’s life caused it to be prioritized over the social area. Moreover, motor dysfunction restricts mobility and thus decreases the quality of life.

Results in the literature show that physical-motor and emotional processing problems have different effects on the quality of life in people with MS [44, 45]. The assessment of disease severity in MS focuses solely on the physical symptoms of the disease. Available data suggest that evaluating a range of cognitive and emotional skills is crucial when measuring functional problems that may arise from MS.

There are some limitations of the present study. First, the fact that the facial emotion recognition tasks used in the study consisted of posed facial expressions instead of natural expressions, unlike real-life situations, may have caused the participants to respond to the empathy quotient subjectively. Second, the fact that the study group included only relapsing–remitting MS patients, this may have resulted in the insufficiency to compare social cognition performance between different MS phenotypes. Another limitation is that the study’s design did not allow us to evaluate the possible changes in social cognition deficits during follow-up interval. Moreover, we evaluated the symptoms of depression by using a self-reported questionnaire instead of a diagnosis by a psychiatrist. Therefore, it would be more appropriate if a more valid method is used to evaluate depression. In addition, we did not consider the cognitive and somatic-affective symptoms of depression which may relate to MS. Further studies that examine whether impaired social cognition is associated with cognitive and somatic-affective symptoms of depression in MS may address this issue.

This study obtains important contributions to the literature about social cognition by including a large group consisting of 120 individuals with MS and 120 healthy controls. We evaluated emotion recognition and empathy and examined their relationship with disease parameters, neuropsychological status, cognition, and quality of life. Moreover, we applied detailed cognitive tests to healthy controls. In addition, we performed FEIT and FEIDT tests to emphasize facial emotion recognition which were significantly impaired in MS patients. The identification of negative emotions was primarily deteriorated among MS patients.

## Conclusions

In conclusion, the present findings showed that facial emotion recognition, discrimination, and empathy deficits are remarkable in MS patients. In light of the data in this study, impaired social cognition in MS patients is related to both cognitive and emotional aspects of the disease. Cognitive decline and social cognition deficits both worsen the quality of life. Therefore, assessing emotion recognition and empathy skills in clinical practice and informing patients may help them to cope with difficulties in social relationships. Such a clinical approach may be beneficial in improving the patients’ quality of life. More studies supported by functional imaging are needed to detect social cognition disorders in MS patients, elucidate their pathogenesis, and reveal their relationship with other cognitive functions.

## Data Availability

Data can be made available on request.

## References

[1] Tuncer N (2006). Cognitive function impairment in multiple sclerosis patients. Turkiye Klin J Med Sci.

[2] Chalah MA, Ayache SS (2017). Deficits in social cognition: an unveiled signature of multiple sclerosis. J Int Neuropsychol Soc.

[3] Bornhofen C, Mcdonald S (2008). Emotion perception deficits following traumatic brain injury: a review of the evidence and rationale for intervention. J Int Neuropsychol Soc.

[4] Spell LA, Frank E (2000). Recognition of nonverbal communication of affect following traumatic brain injury. J Nonverbal Behav.

[5] Phillips LH, Scott C, Henry JD, Mowat D, Bell JS (2010). Emotion perception in Alzheimer’s disease and mood disorder in old age. Psychol Aging.

[6] Ekman P, Friesen WV (1976). Pictures of facial affect.

[7] Kerr SL, Neale JM (1993). Emotion perception in schizophrenia: specific deficit or further evidence of generalized poor performance?. J Abnorm Psychol.

[8] Kurtzke JF (1983). Rating neurologic impairment in multiple sclerosis: an expanded disability status scale (EDSS). Neurology.

[9] Erol A, Unal EK, Gulpek D, Mete L (2009). The reliability and validity of Facial Emotion Identification and Facial Emotion Discrimination Tests in Turkish culture. Anatolian J Psychiatry.

[10] Lawrence EJ, Shaw P, Baker D, Baron-Cohen S, David AS (2004). Measuring empathy: reliability and validity of the Empathy Quotient. Psychol Med.

[11] Aksoy S, Timer E, Mumcu S (2013). Screening for cognitive impairment in multiple sclerosis with MOCA test. Turk J Neurol.

[12] Parmenter BA, Weinstock-Guttman B, Garg N (2007). Screening for cognitive impairment in multiple sclerosis using the Symbol digit Modalities Test. Mult Scler.

[13] Rudick R, Antel J, Confavreux C, Cutter G, Ellison G, Fischer J (1997). Recommendations from the national multiple sclerosis society clinical outcomes assessment task force. Ann Neurol.

[14] Charvet LE, Taub E, Cersosimo B, Rosicki C, Melville P, Krupp LB (2015). The Montreal Cognitive Assessment (MoCA) in multiple sclerosis: relation to clinical features. J Mult Scler.

[15] Beck AT, Steer RA, Brown GK (1996). Manual for the Beck Depression Inventory-II.

[16] Idiman E, Uzunel F, Ozakbas S, Yozbatiran N, Oguz M, Callioglu B (2006). Cross-cultural adaptation and validation of multiple sclerosis quality of life questionnaire (MSQOL-54) in a Turkish multiple sclerosis sample. J Neurol Sci.

[17] Vickrey BG, Hays RD, Harooni R, Myers LW, Ellison GW (1995). A health-related quality of life measure for multiple sclerosis. Qual Life Res.

[18] Cecchetto C, Aiello M, D’Amico D, Cutuli D, Cargnelutti D, Eleopra R (2014). Facial and bodily emotion recognition in multiple sclerosis: the role of alexithymia and other characteristics of the disease. J Int Neuropsychol Soc.

[19] Henry A, Tourbah A, Chaunu MP, Rumbach L, Montreuil M, Bakchine S (2011). Social cognition impairments in relapsing-remitting multiple sclerosis. J Int Neuropsychol Soc.

[20] Prochnow D, Donell J, Schäfer R, Jörgens S, Hartung HP, Franz M (2011). Alexithymia and impaired facial affect recognition in multiple sclerosis. J Neurol.

[21] Krause M, Wendt J, Dressel A, Berneiser J, Kessler C, Hamm AO (2009). Prefrontal function associated with impaired emotion recognition in patients with multiple sclerosis. Behav Brain Res.

[22] Jehna M, Neuper C, Ischebeck A, Loitfelder M, Ropele S, Langkammer C (2011). The functional correlates of face perception and recognition of emotional facial expressions as evidenced by fMRI. Brain Res.

[23] Beatty WW, Goodkin DE, Weir WS, Staton RD, Monson N, Beatty PA (1989). Affective judgments by patients with Parkinson’s disease or chronic progressive multiple sclerosis. Bull Psychon Soc.

[24] Beatty WW, Orbelo DM, Sorocco KH, Ross ED (2003). Comprehension of affective prosody in multiple sclerosis. Mult Scler J.

[25] Banati M, Sandor J, Mike A, Illes E, Bors L, Feldmann A (2010). Social cognition and Theory of Mind in patients with relapsing-remitting multiple sclerosis. Eur J Neurol.

[26] de Almeida MB, Going LC, Fragoso YD (2016). Patients with multiple sclerosis present low levels of empathy. Arq Neuropsiquiatr.

[27] Kraemer M, Herold M, Uekermann J, Kis B, Wiltfang J, Daum I (2013). Theory of mind and empathy in patients at an early stage of relapsing remitting multiple sclerosis. Clin Neurol Neurosurg.

[28] Pitteri M, Genova H, Lengenfelder J, DeLuca J, Ziccardi S, Rossi V (2019). Social cognition deficits and the role of amygdala in relapsing remitting multiple sclerosis patients without cognitive impairment. Mult Scler Relat Disord.

[29] Pakenham KI, Cox S (2009). The dimensional structure of benefit finding in multiple sclerosis and relations with positive and negative adjustment: a longitudinal study. Psychol Heal.

[30] Benedict RH, Priore RL, Miller C, Munschauer F, Jacobs L (2001). Personality disorder in multiple sclerosis correlates with cognitive impairment. J Neuropsychiatry Clin Neurosci.

[31] Gleichgerrcht E, Tomashitis B, Sinay V (2015). The relationship between alexithymia, empathy and moral judgment in patients with multiple sclerosis. Eur J Neurol.

[32] Messina S, Patti F (2014). Gray matters in multiple sclerosis: cognitive impairment and structural MRI. Mult Scler Int.

[33] Charil A, Zijdenbos AP, Taylor J, Boelman C, Worsley KJ, Evans AC (2003). Statistical mapping analysis of lesion location and neurological disability in multiple sclerosis: application to 452 patient data sets. Neuroimage.

[34] Pagani E, Rocca MA, Gallo A, Rovaris M, Martinelli V, Comi G (2005). Regional brain atrophy evolves differently in patients with multiple sclerosis according to clinical phenotype. Am J Neuroradiol.

[35] Berneiser J, Wendt J, Grothe M, Kessler C, Hamm AO, Dressel A (2014). Impaired recognition of emotional facial expressions in patients with multiple sclerosis. Mult Scler Relat Disord.

[36] Dulau C, Deloire M, Diaz H, Saubusse A, Charre-Morin J, Prouteau A (2017). Social cognition according to cognitive impairment in different clinical phenotypes of multiple sclerosis. J Neurol.

[37] Henry JD, Phillips LH, Beatty WW, McDonald S, Longley WA, Joscelyne A (2009). Evidence for deficits in facial affect recognition and theory of mind in multiple sclerosis. J Int Neuropsychol Soc.

[38] Raimo S, Trojano L, Pappacena S, Alaia R, Spitaleri D, Grossi D (2017). Neuropsychological correlates of theory of mind deficits in patients with multiple sclerosis. Neuropsychology.

[39] Ouellet J, Scherzer PB, Rouleau I, Métras P, Bertrand-Gauvin C, Djerroud N (2010). Assessment of social cognition in patients with multiple sclerosis. J Int Neuropsychol Soc.

[40] Pöttgen J, Dziobek I, Reh S, Heesen C, Gold SM (2013). Impaired social cognition in multiple sclerosis. J Neurol Neurosurg Psychiatry.

[41] Batista S, Alves C, D’Almeida OC, Afonso A, Félix-Morais R, Pereira J (2017). Disconnection as a mechanism for social cognition impairment in multiple sclerosis. Neurology.

[42] Palermo R, Rhodes G (2007). Are you always on my mind? A review of how face perception and attention interact. Neuropsychologia.

[43] Realmuto S, Dodich A, Meli R, Canessa N, Ragonese P, Salemi G (2019). Moral cognition and multiple sclerosis: a neuropsychological study. Arch Clin Neuropsychol.

[44] Cotter J, Firth J, Enzinger C, Kontopantelis E, Yung AR, Elliott R (2016). Social cognition in multiple sclerosis: a systematic review and meta-analysis. Neurology.

[45] Phillips LH, Henry JD, Scott C, Summers F, Whyte M, Cook M (2011). Specific impairments of emotion perception in multiple sclerosis. Neuropsychology.

